# Two-Year Data on the Efficacy and Safety of the MINIject Supraciliary Implant in Patients with Medically Uncontrolled Primary Open-Angle Glaucoma

**DOI:** 10.3390/jcm14051639

**Published:** 2025-02-28

**Authors:** Panagiotis Dervenis, Nikolaos Dervenis, Gerassimos Lascaratos, Chrysostomos Dimitriou

**Affiliations:** 1 Moorfields Eye Hospital, Moorfields Eye Hospital NHS Foundation Trust, London EC1V 2PD, UK; 2 Institute of Ageing and Chronic Disease, University of Liverpool, Liverpool L69 7ZX, UK; nikosdervenis@gmail.com; 3 Section of Ophthalmology, School of Life Course Sciences, King’s College London, London SE1 7EH, UK; gerassimos.lascaratos@nhs.net; 4 Colchester Eye Centre, East Suffolk and North Essex NHS Foundation Trust, Colchester CO4 5JR, UK; chr.dimitriou@gmail.com

**Keywords:** glaucoma, intraocular pressure, MIGS, MINIject

## Abstract

**Background/Objectives**: The aim of this study is to evaluate the long-term efficacy and safety of the MINIject supraciliary device in patients with medically uncontrolled primary open-angle glaucoma (POAG) with up to 2 years of follow-up. **Methods**: A retrospective study was conducted using electronic medical records. Patients with medically uncontrolled POAG were included. Patients were either phakic, receiving combined phacoemulsification and MINIject, or pseudophakic receiving standalone MINIject. They were followed up for 24 months postoperatively. The primary outcomes were intraocular pressure (IOP) and use of IOP-lowering medications at 24 months postoperatively. Other secondary outcomes included IOP and use of glaucoma medications at 6, 12, and 18 months, success defined as 6 ≤ IOP ≤ 18 mmHg without the use of medications (complete) or regardless of the use of medications (qualified), average change in visual field (VF) mean deviation (MD) from baseline to 24 months, and adverse events. **Results**: The study included 37 eyes of 37 consecutive glaucoma patients. Of these, 27 underwent combined phacoemulsification with MINIject implantation and 10 had standalone MINIject. The mean preoperative IOP was 17.95 ± 4.75 mmHg on 2.16 ± 1.12 glaucoma drops. At 24 months, the mean IOP was 14.58 ± 4.55 on 0.69 ± 0.98 medications (*p* = 0.002). No difference in IOP was noted between the combined and the standalone group, although the latter had a trend for more glaucoma drops. The qualified success and the complete success rates were 67.6% and 56.8%, respectively. Average VF mean deviation progression was 0.19 ± 1.19 dB. No sight threatening complications occurred. **Conclusions**: MINIject can be a safe and effective alternative to current glaucoma devices, offering significant reduction in IOP and medication use with a good safety profile. Longer term data are needed to confirm its prolonged safety.

## 1. Introduction

Glaucoma is one of the leading causes of irreversible loss of vision, affecting more than 60 million of people worldwide [1]. It represents a form of progressive optic neuropathy, causing loss of visual field and, in the end stages, complete loss of vision. Many risk factors have been associated with glaucoma progression, with the only modifiable being lowering the intraocular pressure (IOP) [2,3].

For many years, topical medications in the form of eye drops have been the mainstay in glaucoma management. Although they are safe and effective in reducing IOP, they present some limitations, mainly regarding compliance issues and side effects, such as ocular surface disease, affecting vision-related quality of life [4,5].

Traditional filtering surgeries, such as trabeculectomy, are still considered the gold standard in glaucoma management and are often performed in combination with cataract surgery. Although highly effective at reducing IOP, they require intensive postoperative follow-up and are associated with potential complications like hypotony, blebitis, and bleb-related interventions. Recently, there has been a shift in the glaucoma treatment paradigm, with the introduction of interventional glaucoma therapy. Selective laser trabeculoplasty and minimally invasive glaucoma surgeries (MIGS) can be offered to patients with early and moderate glaucoma, reducing IOP and medication burden, thus overcoming the compliance issues and limitations of traditional topical medical therapy [5,6,7,8,9,10]. MIGS techniques encompass a range of procedures, including angle surgeries, subconjunctival filtration, and supraciliary drainage, with a wide variety of evolving implants and devices. MIGS enhance aqueous outflow by bypassing the trabecular meshwork, dilating Schlemm’s canal, or diverting the flow to the suprachoroidal space, typically sparing the conjunctiva [11,12].

Despite its potential, the suprachoroidal space remains poorly understood. Over the years, several surgical techniques (such as iatrogenic clefts and setons) and devices like Cypass (Alcon, Geneva, Switzerland) and iStent Supra (Glaukos, San Clemente, CA, USA) have been used to take advantage of this space, but with varied results [13,14,15].

MINIject^®^ (iSTAR Medical SA, Wavre, Belgium) is a new device, made of biocompatible silicone with a porous structure to enhance outflow in the suprachoroidal space. It is implanted via an ab interno procedure, either combined with phacoemulsification or as standalone surgery [16]. Trials have reported a reduction of 40% in IOP at 2 years, with 37.9% of patients being medication-free [9]. However, there is only limited evidence in the literature on real-world data reporting MINIject efficacy and safety in patients with glaucoma. To date, no publications have reported its outcomes as a combined procedure. The aim of our study is to report the two-year, real-world data of MINIject implantation from a single center in the United Kingdom.

## 2. Materials and Methods

### 2.1. Study Design and Participants

A retrospective, single-center, cohort study was conducted using electronic medical records of patients at the Colchester Eye Centre of Excellence, East Suffolk and North Essex NHS Foundation Trust, United Kingdom, in accordance with the principles of the Declaration of Helsinki. Approval by the local Institutional Review Board (IRB) was not required for this retrospective study as it involved the analysis of fully anonymized data, with no identifiable patient information. This study did not involve any direct patient contact, intervention, or changes to standard clinical care. In accordance with local institutional and regulatory guidelines, research using anonymized retrospective data that does not pose risks to patient confidentiality or welfare is typically exempt from formal IRB review. The study was conducted between 1 March 2022 and 31 December 2024. One experienced surgeon (CD) performed all surgeries. No preoperative washout was carried out. Written informed consent was obtained from all participants before surgery.

### 2.2. Inclusion Criteria

Consecutive patients diagnosed with primary open-angle glaucoma (POAG) were enrolled in this study, ensuring a well-defined patient cohort. To qualify for inclusion, participants were required to be at least 18 years of age and have POAG that was not adequately controlled with medical therapy, meaning they were using at least one IOP-lowering medication. The diagnosis of POAG and progression was based on comprehensive assessments, including serial visual field (VF) testing and optical coherence tomography (OCT) scans. Patients demonstrated progressive changes in their visual field defects and retinal nerve fiber layer (RNFL) thinning over at least two consecutive visits, confirming the ongoing disease progression. All surgeries were performed by a single, experienced surgeon (CD) between 1 March 2022, and 31 December 2022. For patients who were already pseudophakic (had previously undergone cataract surgery), the MINIject implantation was performed as a standalone procedure. In contrast, for those who were not pseudophakic, the MINIject implantation was combined with phacoemulsification and intraocular lens implantation, ensuring a comprehensive approach to both the glaucoma treatment and cataract management.

### 2.3. Exclusion Criteria

The exclusion criteria for this study were carefully defined to ensure that the results were not confounded by factors unrelated to the effects of the MINIject implant. Patients with types of glaucoma other than POAG were excluded to focus specifically on the POAG population. Additionally, individuals who experienced intraoperative complications during cataract surgery, if a combined procedure was performed, were excluded to ensure that any adverse outcomes could be attributed solely to the MINIject procedure rather than potential complications arising from cataract surgery. Patients with corneal opacity, which would prevent clear visualization of the angle during gonioscopy, were also excluded, as this would hinder the proper assessment of the surgical site and its effect on IOP. Furthermore, those with clinically significant corneal or retinal disease were excluded, as these conditions could introduce confounding factors affecting the results. Individuals who had undergone prior glaucoma surgery of any kind were excluded to avoid the influence of previous surgical interventions on the outcomes of MINIject implantation. Lastly, patients who had undergone glaucoma laser treatments within 90 days before surgery were excluded, as recent laser treatments could impact baseline IOP measurements and interfere with the assessment of MINIject’s efficacy. These stringent criteria were implemented to ensure the integrity and reliability of the study findings.

### 2.4. Patient Follow-Up

This study subjects were closely monitored for a minimum of 24 months following the implantation procedure to assess the long-term efficacy and safety outcomes of the MINIject (iSTAR Medical SA, Wavre, Belgium) device. This extended follow-up period allowed for a comprehensive evaluation of the device’s performance over time. However, patients who did not complete the full 24-month follow-up were excluded from the study analysis. This included individuals who were lost to follow-up due to reasons such as relocation to a different area or other logistical challenges, as well as those who passed away during the study period. By excluding these cases, we ensured that the analysis was based on a cohort with complete follow-up data, thus maintaining the integrity and reliability of the study’s outcomes.

### 2.5. Study Device and Surgical Implantation Technique

**Figure 1:**
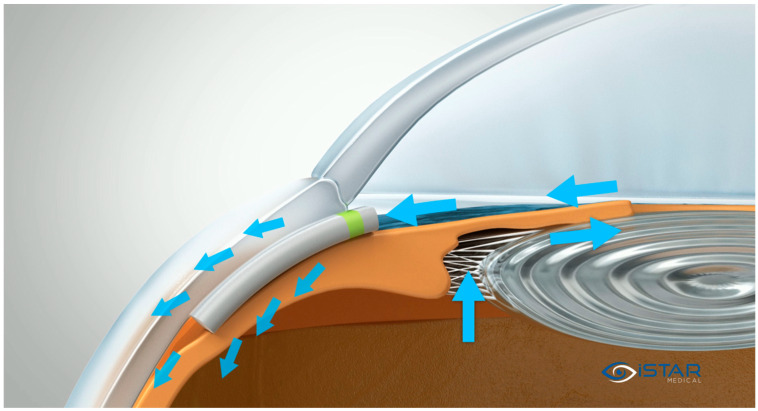
MINIject positioning scheme. The arrows illustrate the pathway of the aqueous humor from production to drainage through the MINIject implant towards the supraciliary space. The image is property of iSTAR Medical SA, Wavre, Belgium, and permission was granted for reproduction.

**Figure 2:**
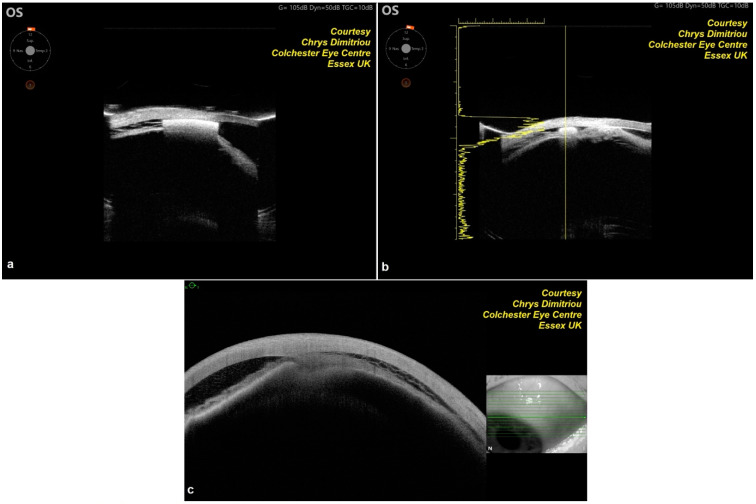
(**a**,**b**) Ultrasound biomicroscopy of MINIject showing a supraciliary cleft and a reservoir of aqueous humor drainage; (**c**) Anterior segment optical coherence tomography of MINIject showing a reservoir of aqueous humor drainage.

The MINIject implant is an innovative, preloaded, ab interno MIGS device specifically designed to improve aqueous humor outflow by directing it into the supraciliary space. Measuring 5 mm in length, the implant is constructed from medical-grade silicone, known as STAR material, which is designed to conform to the anatomical features of the supraciliary space. This material ensures that the device integrates effectively with the surrounding tissue for optimal performance. The MINIject has a unique porous design, which facilitates aqueous drainage through both the small cleft created during implantation and the porous structure of the implant itself. This dual-flow mechanism enhances the efficiency of the implant in reducing IOP. The device is preloaded into a delivery sheath, which is then connected to a delivery tool, making the implantation process straightforward and precise. This design ensures that the device can be accurately placed into the supraciliary space with minimal complication, contributing to its effectiveness as a MIGS option for glaucoma treatment (Figure 1 and Figure 2 illustrate the device and its placement mechanism).

**Figure 3:**
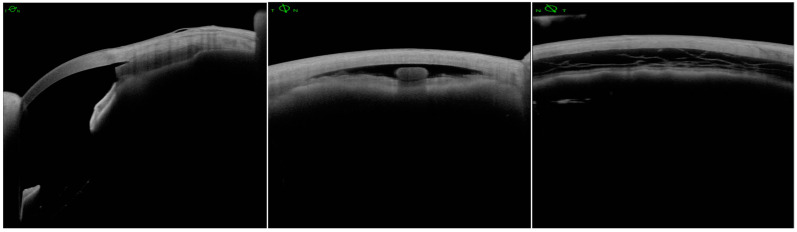
Anterior segment optical coherence tomography sections of MINIject showing a reservoir—supraciliary “bleb”.

**Figure 4:**
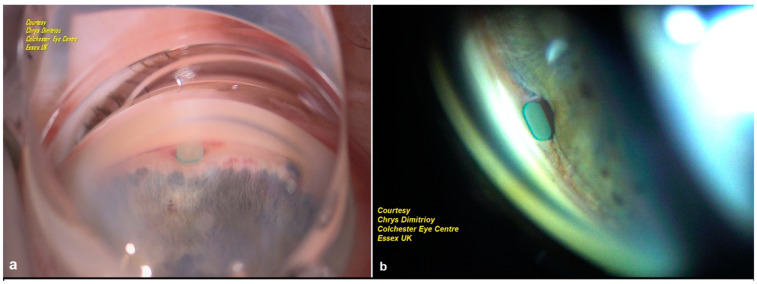
(**a**) Intraoperative gonioscopic image of MINIject immediately after implantation, showing correct positioning with the green ring placed at the scleral spur level. (**b**) Gonioscopic slit lamp image of MINIject implant 6 months after implantation, showing minimal remaining supraciliary cleft and good positioning of the implant away from the corneal endothelium.

The MINIject can be implanted either as a standalone procedure or at the end of cataract surgery. All patients received sub-Tenon’s anesthesia preoperatively, consisting of lignocaine 2% and hyaluronidase 150 IU/mL. A temporal 2.0–2.2 mm clear corneal incision was used. Miochol-E (Bausch & Lomb, Vaughan, ON, Canada) and a highly cohesive viscoelastic (Healon GV Pro, Johnson & Johnson Vision, Jacksonville, FL, USA) were injected to constrict the pupil, form the anterior chamber, and maintain stability. The patient’s head was tilted away from the surgeon as in most MIGS procedures, and a surgical gonioprism was used to visualize the angle. The implant was advanced in the supraciliary space until the green ring was positioned at the level of the scleral spur. This way, only 0.5 mm of the device was exposed in the anterior chamber, thus protecting against corneal endothelium damage (Figure 3 and Figure 4). At the end of the surgery, intracameral cefuroxime 1.0 mg/0.1 mL was given, and the standard postoperative regime included a combination of topical antibiotics and steroids, and, in the case of diabetic patients, a course of topical non-steroidal anti-inflammatory drugs (NSAIDs) was prescribed. All glaucoma medications were discontinued postoperatively, and they were introduced again as needed.

### 2.6. Outcome Measures

The primary outcomes were IOP and use of IOP-lowering medications at 24 months postoperatively. Other secondary outcomes included IOP and use of IOP-lowering medications at 6, 12, and 18 months, success defined as 6 ≤ IOP ≤ 18 mmHg without the use of glaucoma medications (complete success) or regardless of the use of glaucoma medications (qualified success), average change in visual field (VF) mean deviation (MD) from baseline to 24 months, and adverse events. The selection of 18 mmHg as the cut-off for success was based on the results of the Advanced Glaucoma Intervention Study, which found that significant VF progression is unlikely to occur if IOP is kept below 18 mmHg [2].

### 2.7. Statistical Analyses

Data were analyzed using SPSS Statistics for Windows, Version 23.0 (IBM SPSS Statistics for Windows, Version 23.0. Armonk, NY, USA: IBM Corp). Student’s *t*-test and paired *t*-test were used to compare continuous data, and Fisher’s exact test was used to compare categorical data. Survival analyses were conducted to estimate success rates using Kaplan–Meier curves. A *p*-value of less than 0.05 was considered statistically significant.

## 3. Results

This study included 37 eyes of 37 consecutive patients with POAG. Of these, 27 underwent combined phacoemulsification with MINIject implantation and 10 had standalone MINIject. All standalone cases were in pseudophakic eyes. Baseline patient characteristics are summarized in Table 1.

**Figure 5:**
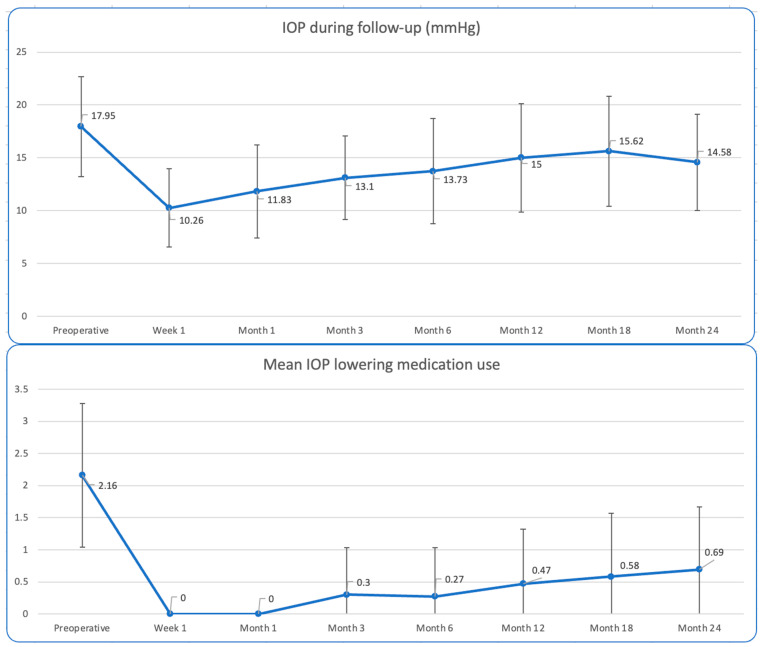
Mean IOP (top panel: X-axis, time; Y-axis, IOP) and mean IOP-lowering medication use (lower panel: X-axis, time; Y-axis, mean number of medications) during follow-up.

Mean preoperative IOP was 17.95 ± 4.75 mmHg on 2.16 ± 1.12 glaucoma drops in the study group. In the combined subgroup the mean preoperative IOP was 18.44 ± 5.03 mmHg on 2.11 ± 1.12 medications, while in the standalone subgroup the mean preoperative IOP was 16.60 ± 3.81 mmHg on 2.3 ± 1.16 mmHg. At 24 months postoperatively, mean IOP was 14.58 ± 4.55 in the study group on 0.69 ± 0.98 medications. This accounts for a statistically significant mean IOP reduction of 3.28 ± 5.78 mmHg (*p* = 0.002) and a statistically significant mean reduction of 1.44 ± 1.13 IOP-lowering medication (*p* < 0.001). At 24 months, 58.3% of operated eyes were medication-free. IOP and medications use during the 24 months follow-up are shown in Table 2 and Figure 5. It is evident that the maximum IOP and glaucoma medications reduction happened during the first 6 months of the follow-up. This is due to drainage both through the created cleft, which tends to scar later, and the implant itself. Additionally, it is visible that SD and IOP reduction values at 3-, 12-, 18- and 24-month time points seem higher than the respective means. No artificial data smoothing or normalization techniques were applied to our data, as we aimed to preserve the real-world nature of our dataset. Given that this study involves real-world data, variability is an inherent aspect of treatment outcomes in glaucoma patients that reflects genuine inter-individual differences.

**Figure 6:**
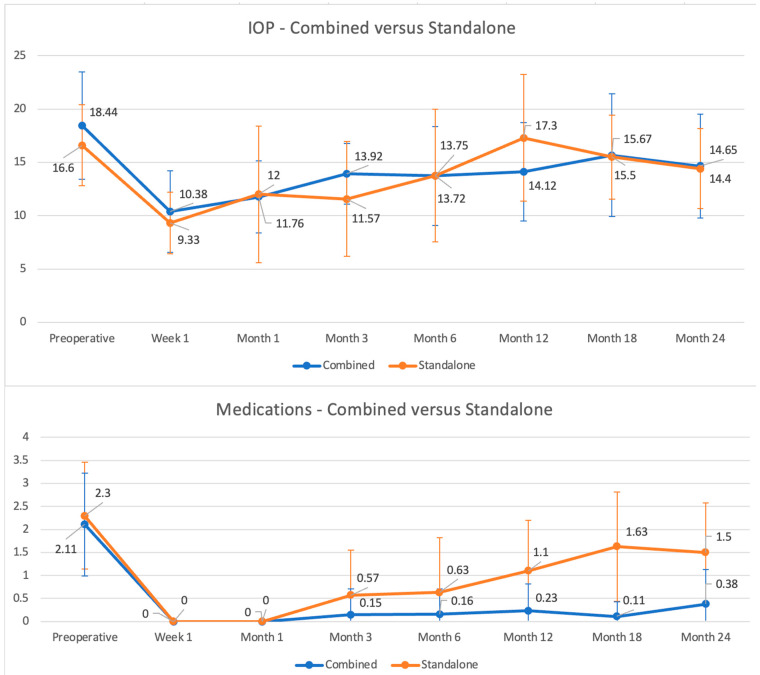
Subgroup analysis of mean IOP (top panel: X-axis, time; Y-axis, IOP) and mean IOP-lowering medication use (lower panel: X-axis, time; Y-axis, mean number of medications) during follow-up.

When performing subgroup analysis based on surgery performed, in the combined subgroup (phacoemulsification/MINIject) the mean IOP at 24 months was 14.65 ± 4.89 mmHg (mean IOP reduction of 3.69 ± 6.13 mmHg, *p* = 0.005) on 0.38 ± 0.75 IOP-lowering medications (mean reduction of 1.69 ± 1.16 medications, *p* < 0.001). In the standalone MINIject subgroup the mean IOP at 24 months was 14.40 ± 3.75 mmHg (mean IOP reduction of 2.20 ± 4.87 mmHg, *p* = 0.04), on 1.50 ± 1.08 medications (mean reduction of 0.80 ± 0.79 medications, *p* = 0.01). When comparing directly the two subgroups, the difference in mean IOP and mean medications use at 24 months did not reach statistical significance (*p*-values were equal to 0.87 and 0.07, respectively), although there was a trend for the combined subgroup to use less medications. Figure 6 shows IOP and medication use during follow-up based on subgroup.

**Figure 7:**
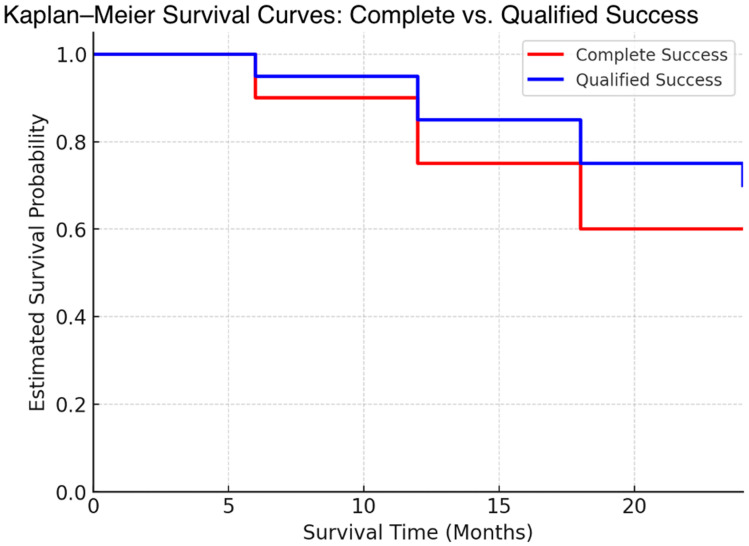
Complete and qualified success Kaplan–Meier survival curves.

Figure 7 shows the Kaplan–Meier survival analyses for complete and qualified success. The qualified success and the complete success rates for the study group at 6 months were 97.3% and 94.6%, respectively. The qualified success and the complete success rates for the study group at 12 months were 89.2% and 73.0%, respectively. The qualified success and the complete success rates for the study group at 18 months were 73.0% and 59.5%, respectively. The qualified success and the complete success rates for the study group at 24 months were 67.6% and 56.8%, respectively.

Regarding VF data, at baseline, MD ± standard deviation (SD) was −5.10 ± 4.62 dB, and at 24 months postoperatively, the average ± SD change in VF MD was 0.19 ± 1.19 dB. This difference was not found to be statistically significant when compared to baseline MD (*p* = 0.55). No patient needed further glaucoma intervention at 2 years.

Adverse events were reported in six eyes, accounting for 16.2% of the cases in our cohort. While this represents a relatively high rate of complications, it is important to emphasize that the majority of these adverse events were transient and self-limiting, with no long-term consequences for patients. Among the reported complications, two eyes developed postoperative hyphema, one of which required a washout procedure. Additionally, cystoid macular edema occurred in two eyes, both of which were managed effectively with the administration of topical NSAIDs, and both cases were in patients who had undergone combined cataract surgery. One patient experienced a trans-zonular spill-over vitreous hemorrhage, which resolved without any medical intervention. Another patient had aqueous misdirection, which was successfully managed during the surgical procedure. Importantly, no sight-threatening complications, such as persistent hypotony, choroidal effusion, or suprachoroidal hemorrhage, were observed in any of the patients. Furthermore, no patients experienced a loss of more than two lines of visual acuity, indicating that the majority of adverse events were not associated with significant or permanent visual impairment.

## 4. Discussion

Our study presents real-world data on the two-year efficacy and safety outcomes of the MINIject implant in patients with POAG. The findings regarding IOP and the number of glaucoma medications required were consistent with the published results from the STAR I-III trials, demonstrating that MINIject’s performance aligns with previously reported data. Notably, our study did not include a washout period, which may have influenced the observed IOP reduction. It can be reasonably assumed that, had a washout period been implemented, the reduction in IOP might have been more pronounced, especially considering that the baseline IOP of some patients in our cohort was relatively low. Additionally, while the STAR trials focused on standalone MINIject procedures, some of the patients in our cohort underwent combined cataract and MINIject surgery. Despite this difference in surgical approach, the mean IOP observed in the combined surgery group was similar to that of the standalone procedure group. However, a trend was noted where patients in the standalone group required more glaucoma medications. While this difference did not reach statistical significance, it is of clinical importance. The enhanced IOP control and medication reduction in the combined group suggest a potential synergistic effect between phacoemulsification and MINIject implantation, which could offer substantial benefits for patients with POAG or cataract co-morbidities. However, it is important to note that the analysis was underpowered, and therefore, this finding requires further validation in a larger sample size to confirm whether the observed trends represent a true effect or are a result of statistical error. Given the clinical implications, a more robust study would be beneficial to fully assess the long-term benefits and reliability of this combined approach in managing IOP and reducing medication use. In our cohort, 58.3% of eyes were medication free, compared to 37.9% reported in the STAR trials. No sight threatening adverse events occurred in this cohort, whereas chorioretinal folds and even papilledema were reported in the company-funded trials [9].

Gläser et al. reported promising results for the MINIject implant, demonstrating its ability to significantly reduce IOP in patients with open-angle glaucoma [17]. However, they included both POAG and pseudoexfoliative glaucoma, thus having a heterogenous sample. Their findings suggest that the device is both safe and effective for suprachoroidal drainage, with minimal complications and a favorable safety profile. These outcomes are consistent with our study, further validating the implant’s role as a treatment option for patients with IOP control issues.

The STAR trials were conducted in order to assess the safety and efficacy of MINIject in OAG patients [9,18,19]. Patients had to have IOP between 21 and 35 mmHg and uncontrolled OAG. A meta-analysis of these studies reported a mean baseline IOP of 23.8 ± 3.3 mmHg on 2.4 ± 1.1 glaucoma medications. The mean VF MD was −6.0 ± 5.5 dB. At 24 months postoperatively, the mean IOP in the pooled group of patients was 14.4 ± 4.5 mmHg on 1.4 ± 1.4 hypotensive medications [9]. However, to the best of our knowledge, there is no study reporting real-world, long-term data.

MIGS devices have shown promising results in terms of IOP-lowering effect and safety profile. Most devices so far aim to enhance aqueous outflow via the conventional pathway. However, this carries the risk of positioning a stent in an area that lacks collector channels, thus reducing efficacy [20]. These devices are also limited by episcleral venous pressure (EVP), not being able to achieve very low pressures. Diverting the aqueous outflow to the supraciliary space has been demonstrated to offer significant pressure reduction, which is unaffected by the positioning of the stent and the EVP [14,21]. The CyPass Microstent (Alcon, Vernier-Geneva, Switzerland) was a MIGS implant that took advantage of the suprachoroidal space. However, the manufacturer withdrew the device from the market due to concerns with long-term endothelial cell loss [22].

Our data compare favorably with published data on other MIGS techniques. Subconjunctival MIGS techniques with the XEN gel stent or the Preserflo microstent are commonly thought to be more effective than angle-based techniques [23]. Most published studies on the XEN gel stent have reported IOP reductions at 12 months between 26.4% and 45.8%. Data on the outcomes of XEN implantation beyond the first year are scarce. One published study reporting 24-month results describes a 27.8% IOP reduction, with similarly stable IOPs beyond 12 months [24]. Results at three years confirmed those from shorter term studies and demonstrate relative stability of the XEN gel stent beyond the first year [25]. In comparison, the Manchester iStent study reported a 19.3% IOP reduction and 38.1% medication reduction 3 years after a single iStent implantation combined with cataract surgery [26]. At present, long-term data on the Preserflo microstent are lacking. Storp et al. reported an overall success rate of 61.9% in a heterogenous group of 160 glaucoma patients after one year follow-up, with 25% of them requiring revision surgery [27]. These results were similar to a multi-center trial from the UK that included 101 eyes with one-year follow-up and showed an overall success rate of 68.1% [28]. A recent meta-analysis comparing trabeculectomy to Preserflo microstent found superior IOP-lowering efficacy and lower rates of secondary surgery with trabeculectomy, while Preserflo had a better safety profile, notably lower rates of hypotony maculopathy [29]. On the other end of the surgical spectrum, the Tube Versus Trabeculectomy trial reported a 39.9% IOP reduction and 32.3% medication reduction 3 years after Baerveldt tube implantation, and a concomitant 49.4% IOP reduction and 62.5% medication reduction in the trabeculectomy group [30]. However, the superior IOP-reduction potential of trabeculectomy, often considered the gold-standard for filtering surgery, is associated with significant complications, with an overall rate of AEs of 60.0%, including 27.0% of serious complications.

When comparing the safety profiles of the Cypass and MINIject devices, both have demonstrated generally favorable outcomes, with most adverse events being transient and manageable [21]. In the Cypass trial, ocular AEs were reported in 39% of the microstent group. Notably, there were instances of transient hypotony and elevated IOP, but these resolved without significant long-term effects. In contrast, in our study, adverse events were reported in 16.2% of eyes, with the majority being self-limiting. These included postoperative hyphema, cystoid macular edema, and a case of vitreous hemorrhage. There were no sight-threatening complications such as persistent hypotony or suprachoroidal hemorrhage, and no patients experienced significant vision loss (≥2 lines of BCVA). As a result, the MINIject device shows a more favorable profile in terms of the absence of more severe complications, which were observed in a subset of Cypass patients during implantation. Overall, both devices exhibit a low incidence of sight-threatening complications, with the MINIject demonstrating a potentially lower risk of more severe postoperative sequelae.

Our study presents long-term, real-world data on the MINIject supraciliary implant, offering valuable insights into its effectiveness and safety over an extended follow-up period. To the best of our knowledge, this study provides the longest follow-up data available on this device, contributing significantly to the body of evidence regarding its performance in clinical practice. A notable strength of this study is the inclusion of VF data following MINIject implantation, as this is an essential aspect of glaucoma management. By evaluating VF changes, we provide a more comprehensive assessment of the device’s impact on both IOP and the preservation of visual function, offering a broader understanding of its long-term clinical outcomes. This combination of extended follow-up and VF data strengthens the reliability of our findings and enhances the overall value of the study.

Despite its strengths, this study also presents some limitations. It is a single arm study, not allowing for comparisons with other glaucoma treatments or devices. Moreover, it is a retrospective study. Another limitation is that endothelial cell count was not investigated in this cohort of patients. However, no patient developed corneal decompensation or signs of corneal edema postoperatively. Of note, CyPass studies did not report significant endothelial cell loss until 5 years of follow-up [22]. As a result, more studies with longer follow-up, investigating the effect of MINIject on the corneal endothelium are needed. Finally, another limitation is that no preoperative washout was performed, thus possibly raising concerns of already low baseline IOP and favorable patient population. However, our study presents a retrospective analysis of real-world data, and we did not perform preoperative medication washout. Landmark trials evaluating the efficacy of other MIGS devices also report low pre-washout IOP [31,32]. In these studies, IOP raised following medication washout, thus causing more profound IOP reduction. It is reasonable to argue that had a washout period been implemented in our study, the baseline IOP might have been even higher and the reduction in IOP more profound, thus producing more impressive results.

## 5. Conclusions

To conclude, the two-year efficacy and safety outcomes observed in this study suggest that the MINIject implant holds considerable promise for patients with glaucoma. The results indicate that MINIject offers effective IOP reduction, along with a favorable safety profile, over the course of two years. Given its ability to reduce IOP while minimizing the risk of severe complications typically associated with more invasive glaucoma surgeries, such as traditional filtration procedures, the MINIject device appears to present a compelling and viable alternative within the spectrum of MIGS. This makes MINIject a potential option for clinicians seeking to provide effective glaucoma management with fewer postoperative risks for their patients.

## Figures and Tables

**Table 1:** Baseline patient characteristics [*n* = 37].

Characteristic	Summary Measure	
	Mean	±SD
Age (years)	80.4	±6.3
VA (logMAR)	0.28	±0.42
IOP (mmHg)	17.95	4.75
IOP-lowering medications	2.16	1.12
Mean deviation (dB)	−5.10	4.62
Sex	*n*	(%)
Male	17	(45.9)
Female	20	(54.1)
Study eye	*n*	(%)
Right	16	(43.2)
Left	21	(56.8)
Surgery performed	*n*	(%)
MINIject and phacoemulsification	27	(73.0)
MINIject standalone *	10	(27.0)

IOP—intraocular pressure; SD—standard deviation; VA—visual acuity. *—all MINIject standalone cases were in pseudophakic eyes.

**Table 2:** IOP and glaucoma medications use during follow-up.

	**Pre-Operative**	**Month 6**	**Month 12**	**Month 18**	**Month 24**
IOP [mean (SD)] mmHg	17.95 (4.75)	13.73 (4.97)	15.00 (5.14)	15.62 (5.19)	14.58 (4.55)
IOP reduction [mean (SD)] mmHg	NA	4.30 (7.85) *	2.86 (7.25)	2.96 (5.43)	3.28 (5.78) *
IOP reduction %, mean	NA	23.96	15.93	16.49	18.27
No. of medications per eye [mean (SD)]	2.16 (1.12)	0.27 (0.76)	0.47 (0.85)	0.58 (0.99)	0.69 (0.98)
Medication-free eyes (%)	0	87.9	72.2	69.2	58.3

IOP—intraocular pressure; NA—not applicable; SD—standard deviation; *—statistically significant.

## Data Availability

Available upon request to correspondence author.
